# Functional and Radiological Outcomes in Minimally Invasive Surgery-Assisted Transforaminal Lumbar Interbody Fusion in Lumbar Degenerative Disease: A Prospective Observational Study

**DOI:** 10.7759/cureus.114771

**Published:** 2026-08-19

**Authors:** Chaitanya Khandelwal, Subrat Mohapatra, Ananta Panda, Udeepto Lodh, Sourav Mishra, Abhinav Sharma, Gyandeep Sarangi, Kshitij Guglani

**Affiliations:** 1 Orthopaedics and Traumatology, Institute of Medical Sciences and SUM Hospital, Bhubaneswar, IND; 2 Department of Orthopaedic Surgery, Medical College Kolkata, Kolkata, IND

**Keywords:** degenerative spine disease, lumbar spinal canal stenosis, minimally invasive spine, minimally invasive surgery-assisted transforaminal lumbar interbody fusion (mis-tlif), transforaminal lumbar interbody fusion (tlif)

## Abstract

Background: Minimally invasive surgery-assisted transforaminal lumbar interbody fusion (MIS-TLIF) is increasingly used for the surgical management of selected patients with degenerative lumbar spine disease. By utilizing a muscle-sparing approach, MIS-TLIF aims to achieve neural decompression and spinal stabilization while reducing approach-related morbidity. This study evaluated the functional and radiological outcomes of MIS-TLIF in patients with degenerative lumbar disease.

Methods: A prospective observational study was conducted at Institute of Medical Sciences and SUM Hospital, Bhubaneswar, India, on 30 consecutive patients with lumbar degenerative disease who had failed at least six months of conservative treatment. Patients included in the study had unilateral lower-limb radiculopathy associated with radiologically confirmed degenerative lumbar pathology. All patients underwent single-level unilateral MIS-TLIF using a tubular retractor system, local autologous bone graft, PEEK (polyetheretherketone) or titanium interbody cage, and pedicle screw fixation. Intraoperative localization and implant positioning were performed using fluoroscopy; navigation and routine neuromonitoring were not used. Clinical outcomes were assessed using the Visual Analog Scale (VAS) and Oswestry Disability Index (ODI). Radiological assessment included intervertebral disc height, foraminal height, segmental stability, and fusion status. Patients were followed for six months postoperatively.

Results: Thirty patients were included, with a mean age of 51.7 ± 2.3 years (range, 40-62 years). Seventeen procedures were performed at L4-L5 and 13 at L5-S1. Significant postoperative improvement was observed in VAS and ODI scores at all follow-up intervals compared with baseline (p < 0.001). The mean VAS decreased from 7.67 ± 1.09 preoperatively to 1.03 ± 0.72 at six months, while the mean ODI decreased from 63.8 ± 7.51 to 11.5 ± 2.79. Intervertebral disc height increased from 6.88 ± 1.36 mm to 12.1 ± 1.28 mm at two weeks and remained stable at six months. Foraminal height increased from 13.7 ± 1.88 mm to 18.8 ± 1.42 mm at two weeks and remained 18.6 ± 1.42 mm at six months. Fusion was demonstrated in nine of 30 patients (30%) at six months according to the Modified Bridwell criteria on plain radiographs. The mean operative time was 178 ± 38.9 minutes, estimated blood loss was 139 ± 18.2 mL, and mean hospital stay was 3.33 ± 0.99 days. Recorded complications included dural tear, pedicle screw malposition, and postoperative hematoma.

Conclusions: MIS-TLIF demonstrated favorable short-term clinical and radiological outcomes in this prospective cohort. However, the small sample size, six-month follow-up, absence of a comparator group, lack of blinded outcome assessment, and plain-radiograph-based fusion assessment limit conclusions regarding definitive long-term fusion and comparative efficacy. Larger studies with longer follow-up and appropriate comparator groups are required.

## Introduction

Degenerative disorders of the lumbar spine are a major cause of morbidity and disability and may present with axial low-back pain, radiculopathy, sensory symptoms, motor deficits, and impaired ambulation [1,2]. The pathological processes may include disc degeneration, facet arthropathy, ligamentous hypertrophy, and segmental instability. When neural compression is present, decompressive procedures, such as foraminotomy, discectomy, or laminectomy, may relieve radicular symptoms; however, decompression alone may not address structural instability or other mechanical sources of symptoms [3].

Lumbar spinal fusion is an established surgical treatment for appropriately selected patients with degenerative lumbar pathology. Available techniques include posterolateral fusion, posterior lumbar interbody fusion (PLIF), anterior lumbar interbody fusion (ALIF), lateral lumbar interbody fusion, and transforaminal lumbar interbody fusion (TLIF). TLIF was developed as an alternative posterior interbody fusion technique and provides unilateral access to the disc space while limiting direct neural retraction [4,5].

Conventional open lumbar fusion requires extensive paraspinal muscle dissection and retraction, which may contribute to postoperative pain, muscle injury, and delayed recovery [6,7]. Minimally invasive surgery (MIS)-TLIF was developed to achieve the objectives of decompression and interbody fusion through a muscle-sparing tubular approach [8]. Previous studies have reported favorable clinical outcomes, reduced blood loss, shorter hospital stay, and satisfactory radiological outcomes following MIS-TLIF [9-11].

The present study was undertaken to evaluate the short-term functional and radiological outcomes of MIS-TLIF in a prospectively followed cohort of patients with degenerative lumbar disease. The study specifically assessed pain, disability, disc height, foraminal height, segmental stability, and early radiographic fusion.

## Materials and methods

​​​​​​Study design and setting

This was a prospective observational study conducted over one year at the Institute of Medical Sciences and SUM Hospital, a tertiary care center in Bhubaneswar, India. The primary objective was to evaluate functional and radiological outcomes following MIS-TLIF in patients with degenerative lumbar disease. Eligible patients presenting during the study period were consecutively assessed for inclusion.

Ethical considerations

The study was approved by the Institutional Ethics Committee of the Institute of Medical Sciences and SUM Hospital before initiation (ref. no/IEC/IMS.SH/SOA/2024/696). Written informed consent was obtained from all participants. Clinical and outcome data were collected prospectively in accordance with ethical research standards.

Inclusion and exclusion criteria

Patients aged 18 years or older with radiologically confirmed degenerative lumbar disease and unilateral lower-limb radiculopathy who had failed at least six months of conservative management were eligible for inclusion. Patients were required to provide informed consent and comply with the follow-up protocol.

The final cohort consisted exclusively of patients undergoing single-level MIS-TLIF. Cases requiring fusion at more than one level were excluded from the final study cohort. The study was therefore restricted to single-level procedures to maintain a relatively homogeneous operative population and reduce heterogeneity in operative complexity and postoperative outcomes.

Other exclusion criteria included active systemic or local infection, previous fusion surgery at the affected level, severe osteoporosis or other conditions likely to impair fusion, malignancy, immunosuppressive disorders, pregnancy, cognitive impairment affecting follow-up or outcome assessment, refusal to provide consent, and known hypersensitivity to implant materials.

Patients with bilateral radiculopathy, isolated axial low-back pain without radicular symptoms, or neurogenic claudication/canal stenosis without documented instability were not included as separate predefined clinical subgroups and were not analyzed separately.

Preoperative assessment

All patients underwent clinical evaluation including demographic profiling, medical history, symptom assessment, and neurological examination. Preoperative imaging included standing anteroposterior and lateral radiographs, dynamic flexion-extension radiographs, CT, and MRI of the lumbosacral spine. Routine laboratory investigations were performed as appropriate.

Functional and radiological evaluation

Functional assessment was performed preoperatively and at two weeks, six weeks, three months, and six months postoperatively using the Visual Analog Scale (VAS) for pain and Oswestry Disability Index (ODI) for functional impairment.

Postoperative radiological assessment was performed using plain radiographs, including standing radiographs and dynamic flexion-extension radiographs. Intervertebral disc height, neural foraminal height, and segmental stability were assessed. Spondylolisthesis was graded according to the Meyerding classification.

Fusion was assessed on plain radiographs according to the modified Bridwell criteria. CT was not routinely used for postoperative fusion assessment. Therefore, the six-month fusion findings represent an early radiographic assessment and should not be considered definitive CT-confirmed arthrodesis.

For the purposes of this study, successful radiographic fusion was defined according to the applicable modified Bridwell criteria, based on radiographic evidence of interbody bridging and the absence of findings indicating radiographic nonunion.

Surgical technique

All patients underwent a unilateral MIS-TLIF procedure. The patient was positioned prone, and the operative level was confirmed using intraoperative fluoroscopy.

A tubular retractor system (Medtronic) was used to obtain minimally invasive access to the operative level. Following tubular exposure, the facet joint was approached, and the required facetectomy and neural decompression were performed. The disc space was subsequently accessed through the transforaminal corridor.

Disc material was removed, and the endplates were prepared for interbody fusion. Local autologous bone obtained during the decompression was used as the bone graft material. A PEEK (polyetheretherketone) or titanium interbody cage was inserted into the prepared disc space. Pedicle screw fixation was subsequently performed using a Medtronic instrumentation system.

Implant positioning was confirmed intraoperatively using fluoroscopy. Computer-assisted navigation was not used, and routine intraoperative neuromonitoring was not performed.

Follow-up protocol

Patients were followed clinically and radiologically in the immediate postoperative period and at two weeks, six weeks, three months, and six months after surgery.

Outcome measures

The primary outcomes were changes in VAS and ODI scores from baseline to each postoperative time point, together with changes in intervertebral disc height, foraminal height, and segmental stability.

Secondary outcomes included early radiographic fusion, perioperative and postoperative complications, operative duration, estimated blood loss, and hospital stay.

Statistical analysis

Data were analyzed using IBM SPSS Statistics for Windows, version 26.0 (IBM Corp., Armonk, NY, USA). Descriptive statistics were used to summarize demographic, clinical, radiological, and perioperative variables. Paired comparisons were used to evaluate changes in continuous variables between baseline and postoperative assessments. A p-value of <0.05 was considered statistically significant.

Because multiple postoperative time points were evaluated, repeated comparisons may increase the risk of type I error. No formal adjustment for multiple comparisons was performed in the original analysis. Given the small sample size and exploratory observational design, the statistical findings are interpreted primarily as evidence of temporal improvement rather than definitive comparative treatment efficacy.

No formal prior power calculation was performed because the study was designed as a prospective observational cohort with consecutive recruitment during a predefined study period.

## Results

Patient characteristics

Thirty patients were included in the study, comprising 18 males and 12 females. The mean age was 51.7 ± 2.3 years, with a range of 40-62 years. Seventeen patients underwent MIS-TLIF at L4-L5 and 13 at L5-S1. All patients underwent single-level procedures.

Clinical outcomes

Table 1 demonstrates a statistically significant improvement in both VAS and ODI scores following surgery. 

**Table 1:** Clinical outcome scores over time (VAS and ODI) VAS: Visual Analog Scale; ODI: Oswestry Disability Index; SD: standard deviation

Time point	VAS score (mean ± SD)	VAS median	VAS range	ODI score (mean ± SD)	ODI median	ODI range	p-value
Preoperative	7.67 ± 1.09	8.0	5–9	63.8 ± 7.51	65.0	46–76	Reference
Two weeks postoperative	2.73 ± 0.74	3.0	1–4	25.8 ± 5.06	26.0	16–36	< 0.001
Six weeks postoperative	2.20 ± 0.66	2.0	1–3	20.7 ± 5.01	20.0	14–32	< 0.001
Three months postoperative	1.73 ± 0.69	2.0	0–3	16.6 ± 3.13	16.0	11–24	< 0.001
Six months postoperative	1.03 ± 0.72	1.0	0–2	11.5 ± 2.79	11.0	7–20	< 0.001

The mean preoperative VAS and ODI scores were 7.67 ± 1.09 and 63.8 ± 7.51, respectively. Both scores decreased progressively following surgery.

At two weeks, the mean VAS was 2.73 ± 0.74, and the mean ODI was 25.8 ± 5.06. At six weeks, the corresponding values were 2.20 ± 0.66 and 20.7 ± 5.01. At three months, VAS was 1.73 ± 0.69, and ODI was 16.6 ± 3.13. At six months, VAS was 1.03 ± 0.72, and ODI was 11.5 ± 2.79. All postoperative values were significantly improved compared with baseline (p < 0.001).

The reduction in ODI from 63.8 preoperatively to 25.8 at two weeks represented an absolute improvement of 38.0 points. Because this was a non-randomized study without a control group or blinded outcome assessment, the magnitude of early patient-reported improvement should be interpreted cautiously.

Radiological outcomes

Intervertebral disc height increased from 6.88 ± 1.36 mm preoperatively to 12.1 ± 1.28 mm at two weeks and remained 12.1 ± 1.28 mm at six months (p < 0.01), as demonstrated in Table 2.

Foraminal height increased from 13.7 ± 1.88 mm preoperatively to 18.8 ± 1.42 mm at two weeks and remained 18.6 ± 1.42 mm at six months (p < 0.01).

Fourteen patients had preoperative segmental instability. Resolution of instability was observed postoperatively and remained stable during the follow-up period.

Radiographic fusion according to the modified Bridwell criteria was demonstrated in 9 of 30 patients (30%) at six months. Because fusion was assessed using plain radiographs rather than CT, this represents an early radiographic fusion assessment and may not reflect definitive long-term arthrodesis.

**Table 2:** Radiological and perioperative parameters mm: millimeter; mL: milliliter; min: minutes; SD: standard deviation

Parameter	Preoperative (mean ± SD)	Postoperative (two weeks)	Postoperative (six months)	p-value
Intervertebral disc height (mm)	6.88 ± 1.36	12.1 ± 1.28	12.1 ± 1.28	< 0.01
Foraminal height (mm)	13.7 ± 1.88	18.8 ± 1.42	18.6 ± 1.42	< 0.01
Operative duration (min)	178 ± 38.9	–	–	–
Blood loss (mL)	139 ± 18.2	–	–	–
Hospital stay (days)	3.33 ± 0.99	–	–	–
Fusion at six months (%)	–	–	30% (9/30 patients)	–

Operative and perioperative outcomes

The mean operative duration was 178 ± 38.9 minutes. The mean estimated blood loss was 139 ± 18.2 mL, and the mean hospital stay was 3.33 ± 0.99 days.

Complications

The recorded complications included dural tear in one patient, pedicle screw malposition in two patients, and postoperative hematoma in one patient. These complications were documented during the perioperative or postoperative follow-up period.

## Discussion

TLIF was developed as an alternative posterior interbody fusion technique and provides access to the disc space through a unilateral transforaminal corridor while minimizing direct neural retraction [12,13]. Compared with conventional posterior approaches, the minimally invasive technique aims to preserve posterior musculo-ligamentous structures and reduce approach-related tissue injury. 

In the present cohort of 30 patients, significant improvement was observed in both VAS and ODI scores at all postoperative time points. The mean VAS decreased from 7.67 preoperatively to 1.03 at six months, while the ODI decreased from 63.8 to 11.5. These findings demonstrate substantial short-term improvement in pain and disability.

The marked improvement in the ODI at two weeks deserves cautious interpretation. The mean ODI decreased by 38 points from baseline. Although this represents substantial early improvement, the study was non-randomized and did not include a control group or blinded outcome assessment. Therefore, response bias, regression to the mean, placebo effects, and other nonspecific influences cannot be excluded.

Radiologically, intervertebral disc height increased from 6.88 mm preoperatively to 12.1 mm at two weeks and remained stable at six months. Foraminal height similarly increased from 13.7 mm to 18.8 mm at two weeks and remained 18.6 mm at six months.

Fusion was demonstrated in 30% of patients at six months according to the modified Bridwell criteria. Although this rate appears relatively low compared with some previously published MIS-TLIF series, the finding should be interpreted cautiously. Interbody fusion is a progressive biological process, and six months may be too early to determine the final fusion status. Furthermore, fusion in this study was assessed using plain radiographs rather than CT. Consequently, the six-month value should be regarded as an early radiographic fusion finding rather than definitive long-term arthrodesis.

The distribution of operative levels was 17 cases at L4-L5 and 13 at L5-S1. Although level-specific factors may affect surgical difficulty, the small sample size of each subgroup limits meaningful inferential comparison between L4-L5 and L5-S1 in the present study.

Reference [3] provides guideline-level support for fusion procedures in appropriately selected patients with degenerative lumbar pathology. We have therefore emphasized that the indication for fusion in appropriately selected degenerative disease is supported by published guideline-level evidence.

This study has several important limitations. First, the sample size was small, with only 30 patients. No formal a priori power calculation was performed because the study was designed as a prospective observational cohort with consecutive recruitment during a predefined study period. The small sample limits statistical power and generalizability.

Second, follow-up was limited to six months. This duration allows assessment of early clinical recovery and early radiological changes but is insufficient to establish definitive long-term fusion, durability of functional improvement, implant stability, or late complications.

Third, postoperative fusion was assessed using plain radiographs rather than CT. Therefore, the six-month fusion rate should be interpreted as an early radiographic finding and not as definitive CT-confirmed fusion.

Fourth, the study did not include a comparator group. Consequently, MIS-TLIF could not be directly compared with open TLIF or continued conservative treatment, limiting causal inference and preventing conclusions regarding superiority.

Fifth, outcome assessment was not blinded. This may introduce observer or measurement bias, particularly in patient-reported and radiological outcomes.

Sixth, the study was conducted at a single institution and reflects the experience of a specific surgical team. Institutional practices and surgeon-specific factors may therefore limit external validation and generalizability.

Finally, the cohort consisted of selected patients with unilateral lower-limb radiculopathy undergoing single-level MIS-TLIF. The findings may not be generalizable to patients requiring multilevel fusion or to other clinical presentations of degenerative lumbar disease.

Future studies should include larger multicenter cohorts, longer follow-up of at least 12 months and preferably longer, standardized CT-based assessment of fusion, blinded outcome assessment where feasible, and matched or randomized comparator groups.

## Conclusions

MIS-TLIF demonstrated favorable short-term clinical and radiological outcomes in selected patients with degenerative lumbar disease. Significant improvement was observed in pain and disability scores, with restoration and maintenance of intervertebral disc and foraminal height.

However, the small sample size, six-month follow-up, absence of a comparator group, lack of blinded outcome assessment, single-center design, and plain-radiograph-based fusion assessment limit conclusions regarding definitive long-term fusion and comparative efficacy. The 30% fusion rate at six months should therefore be interpreted as an early radiographic finding rather than definitive long-term fusion failure.

Larger multicenter prospective studies with follow-up of at least 12 months and preferably longer, CT-based fusion assessment, and appropriate comparator groups are required to evaluate sustained fusion, durability of functional improvement, and late complications.

## References

[1] Deyo RA, Mirza SK, Martin BI (2006). Back pain prevalence and visit rates: estimates from U.S. national surveys, 2002. Spine (Phila Pa 1976).

[2] Katz JN (2006). Lumbar disc disorders and low-back pain: socioeconomic factors and consequences. J Bone Joint Surg Am.

[3] Resnick DK, Choudhri TF, Dailey AT (2005). Guidelines for the performance of fusion procedures for degenerative disease of the lumbar spine. Part 7: intractable low-back pain without stenosis or spondylolisthesis. J Neurosurg Spine.

[4] Harms J, Rolinger H (1982). [A one-stager procedure in operative treatment of spondylolistheses: dorsal traction-reposition and anterior fusion (author's transl)]. Z Orthop Ihre Grenzgeb.

[5] CL RB (1953). The treatment of ruptured lumbar intervertebral discs by vertebral body fusion. I. Indications, operative technique, after care. J Neurosurg.

[6] Kim KT, Lee SH, Suk KS, Bae SC (2006). The quantitative analysis of tissue injury markers after mini-open lumbar fusion. Spine (Phila Pa 1976).

[7] Mayer H (2011). Minimally invasive spine surgery: a surgical manual.

[8] Foley KT, Lefkowitz MA (2002). Advances in minimally invasive spine surgery. Clin Neurosurg.

[9] Wang MY, Mummaneni PV (2010). Minimally invasive surgery for thoracolumbar spinal deformity: initial clinical experience with clinical and radiographic outcomes. Neurosurg Focus.

[10] Peng CW, Yue WM, Poh SY, Yeo W, Tan SB (2009). Clinical and radiological outcomes of minimally invasive versus open transforaminal lumbar interbody fusion. Spine (Phila Pa 1976).

[11] Villavicencio AT, Burneikiene S, Roeca CM, Nelson EL, Mason A (2010). Minimally invasive versus open transforaminal lumbar interbody fusion. Surg Neurol Int.

[12] Harms JG, Jeszenszky D (1998). The unilateral transforaminal approach for posterior lumbar interbody fusion. Orthop Traumatol.

[13] Ray CD (1997). Threaded titanium cages for lumbar interbody fusions. Spine (Phila Pa 1976).

